# Editorial: Non-coding RNAs in Reproductive Biology

**DOI:** 10.3389/fcell.2021.712467

**Published:** 2021-06-24

**Authors:** Li Meng, Katja Teerds, Zhonglin Tang, Bo Zuo, Linjun Hong

**Affiliations:** ^1^National Engineering Research Center for Breeding Swine Industry, College of Animal Science, South China Agricultural University, Guangzhou, China; ^2^Department of Obstertrics and Gynecology, Faculty of Medicine, The Chinese University of Hong Kong, Shatin, China; ^3^Human and Animal Physiology, Wageningen University, Wageningen, Netherlands; ^4^Shenzhen Branch, Guangdong Laboratory for Lingnan Modern Agriculture, Agricultural Genomics Institute at Shenzhen, Chinese Academy of Agricultural Sciences, Shenzhen, China; ^5^Kunpeng Institute of Modern Agriculture at Foshan, Foshan, China; ^6^Key Laboratory of Agriculture Animal Genetics, Breeding and Reproduction of the Ministry of Education, Huazhong Agricultural University, Wuhan, China; ^7^Guangdong Provincial Key Laboratory of Agro-Animal Genomics and Molecular Breeding, College of Animal Science, South China Agricultural University, Guangzhou, China

**Keywords:** ncRNAs, reproduction, embryo, development, epigenetic regulation

Non-coding RNAs (ncRNAs) consist of several classes of transcripts that can be classified according to their function into housekeeping (e.g., ribosomal RNA and transfer RNA) and regulatory RNAs. The main categories of regulatory RNAs include microRNAs (miRNAs), small interfering RNAs (siRNAs), PIWI-interacting RNAs (piRNAs), long non-coding RNAs (lncRNAs) and circular RNAs (circRNAs). ncRNAs are considered as master regulators of transcription, transcript stability, and post-transcriptional and epigenetic regulators of protein-coding transcripts, with functions in both physiological processes and human diseases. To date, much emphasis in research on reproductive biology is put on the identification of protein-coding genes that play important roles in the development of organs and tissues involved in reproduction. Mutations in these genes can have devastating effects on the reproductive system. Surprisingly, much less attention is paid to the possible importance of ncRNAs in reproduction. Several examples in reproductive physiology and disease clearly demonstrate that ncRNAs are essential for proper cellular interactions such as ovarian follicle and embryo development, and that alterations in ncRNAs may play a role in various diseases such as ovarian and prostate cancer.

The Research Topic on “Non-Coding RNAs in Reproductive Biology” in *Frontiers in Cell and Developmental Biology* includes a series of 9 articles that discuss recent advances regarding the biological and molecular roles of ncRNAs in reproductive processes and highlight challenges and outstanding questions, that need to be addressed in future researches.

The gonadal organs testis and ovary function not only as gamete producers but also as endocrine organs involved in the synthesis of sex steroid hormones which are crucial for successful reproduction. In the testis, somatic Sertoli cells directly support and nurse male germ cells in different stages of development, thus playing an essential role in spermatogenesis. Liu et al. explored the expression, function, and mechanism of action of human miR-100-3p in Sertoli cell development, using molecular and physiological methods. The authors demonstrate that miR-100-3p is more highly expressed in the Sertoli cells cultured in the presence of 10% fetal bovine serum (FBS) than 0.5% FBS, promoting DNA synthesis and cell proliferation, while reducing apoptosis simultaneously. Through a loss of function study, authors prove that miR-100-3p directly binds the downstream target gene, serum/glucocorticoid regulated kinase family member 3 (*SGK3*), which main function is related to stimulation of Sertoli cell development. Next to miRNAs, another new type of ncRNAs, circRNAs with closed loop structure, have been reported to play an important role in ovarian follicular development. Li et al. recently identified an N6-methyladenosine (m6A)-modified circRNA, circGFRa1, which is abundantly expressed in the mouse ovary and stage-specifically expressed in cells that are by the authors considered to be mouse female germline stem cells (FGSCs). The authors demonstrate that silencing circGFRa1 in FGSCs significantly reduces their self-renewal capabilities, while overexpression of circGFRa1 significantly enhances FGSC self-renewal. Furthermore, authors show that circGFRa1 can enhance the expression of the downstream target GFRa1, leading to activation of the glial cell derived neurotrophic factor (GDNF) signaling pathway by sponging miR-449 in FGSC. Although these data are very exciting, some caution is necessary, as the presence of FGSC in the postnatal (adult) mammal ovary continues to be under debate.

Embryo implantation failure is recognized as a leading cause of infertility. The review by Zhou and Dimitriadis surveys a group of secreted miRNAs, that are potentially useful in predicting implantation outcome using materials that can be collected in a relatively non-invasive way, such as follicular fluid, blood and uterine fluid. Secreted or extracellular miRNAs are highly stable in body fluids and can be used as a reflection of disease state. A further advantage of these miRNAs is that they are easily detectable in a short time frame. These advantages make them promising biomarkers for the detection of (successful) embryo implantation.

The placenta is the structure where exchange of blood borne factors between mother and fetus occur and as such plays a central role in maternal and fetal health during pregnancy. Xu et al. in their mini-review address the role of placenta-derived miRNAs in the pathophysiology of human pregnancy. miRNAs produced by the maternal site of the placenta can be selectively incorporated into exosomes and potentially transferred into fetal cells to provide intercellular communication between mother and fetus. In this review, the authors especially focus on the role of exosome miRNAs as possible biomarkers for the prediction of pregnancy related diseases, such as preeclampsia.

Next to embryo implantation, proper trophoblast invasion and fusion are also pivotal processes for the establishment of a successful pregnancy. Two original research papers discuss the emerging findings that non-coding RNAs are involved in regulating trophoblast differentiation, invasion, and fusion. You et al. delineated the precise molecular physiological processes by which BMP2 regulates trophoblast invasion. The authors show that serum BMP2 concentrations are significantly lower in women with early pregnancy loss than in women with an ongoing early pregnancy. In mice, exogenous BMP2 promotes embryonic development by stimulating blastocyst formation and hatching. Using primary extravillous trophoblast cells, the authors observed that BMP2 upregulates the downstream LncRNA NR026833.1, promoting SNAIL expression via sponging miR-502-5p. SNAIL then enhances MMP2 expression and promotes cell invasion. Apicella et al. identified two non-coding transcripts, miR-193b and lncRNA UCA1, in the BeWo trophoblast cell model and in placental diseases. The authors show that miR-193b is a hub for the downregulation of 135 targets genes mainly involved in cell cycle progression and energy usage/nutrient transport. Furthermore, it is reported that UCA1 knockdown leads to an altered gene expression profile which may affect trophoblast cell fusion.

Successful reproduction is highly dependent on the health status of the reproductive organs. Finding cures for diseases such as ovarian and prostate cancer is of the utmost importance not only from a reproductive perspective but also from the point of human well-being. Takeiwa et al. provide an outstanding review of the regulatory effects of some lncRNAs in apoptosis of ovarian cancer cells. In particular the authors focus on the molecular characteristics of apoptosis-related lncRNAs, involved in the regulation of transcription factors, histone modification complexes, miRNAs, and protein stability. The authors provide insight in the possible role of apoptosis-related lncRNAs as biomarkers for ovarian cancer diagnosis, prognosis, and therapy. In line with the review by Takeiwa et al.; Hu et al. execute an extensive integrated bioinformatic analysis to characterize lncRNA-immune interactions in prostate cancer. Prostate cancer-specific dysregulated lncRNAs such as RP11-627G23.1 and RP11-465N4.5 are identified and seem to be closely associated with immune-related hallmarks of prostate cancer.

At last, we are pleased with the article by Luo et al. investigating the regulatory roles of non-coding RNAs in fetal and postnatal muscle development. The focus of this study is on fetal development with the emphasis on muscle development as part of fetal well-being. In this review, the authors provide an up-to-date research overview of miRNAs, circRNAs, and lncRNAs involved in regulating myoblast proliferation, differentiation, and postnatal muscle development through multiple molecular mechanisms.

In conclusion, with this Research Topic we have collected a series of reviews and original research papers describing the role of non-coding RNAs in both physiological and pathological processes related to male and female reproduction. The papers comprising this Research Topic greatly contributed to our further understanding of the regulatory mechanisms and potential applications of ncRNAs, although we also learnt that many questions remain still unanswered.

## Author Contributions

LM and LH drafted and further revised the manuscript. KT critically reviewed the manuscript and significantly improved it. ZT and BZ have critically reviewed it. All authors listed have read this editorial and approved it for publication.

## Conflict of Interest

The authors declare that the research was conducted in the absence of any commercial or financial relationships that could be construed as a potential conflict of interest.

